# A community outreach program enhances education on opioid and e-cigarette misuse among teenagers

**DOI:** 10.3389/fpubh.2025.1490166

**Published:** 2025-04-08

**Authors:** Basma M. Klump, Ananya Varre, John P. Karns, Esha Garg, Richa Patel, Urja Parikh, Anthony Mansour, Carrie Nazaroff, Carolina Restini

**Affiliations:** ^1^College of Osteopathic Medicine, Michigan State University, East Lansing, MI, United States; ^2^Detroit Medical Center, Michigan State University, Detroit, MI, United States; ^3^Macomb University College, Michigan State University, Clinton Township, MI, United States

**Keywords:** substance use epidemics, substance misuse, high-school students, medical education, health literacy, community services, opioids awareness, e-cigarettes awareness

## Abstract

**Introduction:**

Substance use epidemics, particularly opioids and e-cigarettes, pose a significant public health crisis, especially among minors. To address opioid and e-cigarette epidemics among young individuals, the Substance Use Prevention (SUP) program educated high school students in southeast Michigan. Through a medical student-led intervention, we implemented interactive educational methods to deliver evidence-based information on the risks associated with these substances.

**Methods:**

A *non-randomized pre- and post-test* quasi-experimental study design assessed the impact of the SUP interventions on the high school students' understanding of addiction mechanisms, health consequences, and prevention strategies. We assessed baseline knowledge (pre-intervention questionnaires), demographic factors, and post-intervention knowledge.

**Results:**

Data analysis among 100 students from four high schools revealed that while students started with varied levels of baseline knowledge, they reported significantly higher confidence in their opioid (*p* < 0.0001) and e-cigarette (*p* < 0.0001) knowledge after the intervention. Students significantly (*p* < 0.05) improved their ability to recognize causes of overdose (scoring 65% vs. 78%), risk factors (21%–84%), and naloxone as emergency treatment (38%–80%) after the intervention.

**Discussion:**

Subjects showed no changes in individual knowledge of e-cigarettes, which we attribute to school-specific variances and/or high baseline knowledge. The difference in knowledge among schools may be due to disparities in race and differences in socioeconomic status, as shown by the increased poverty level. This study evidenced the importance of raising awareness among adolescents to improve their learning and comprehension of the causes and consequences of substance misuse by sharing medically focused explanations of substances as well as the economic and societal impact.

## 1 Introduction

Substance use epidemics, including opioids and e-cigarettes, have become a public health crisis for the community, particularly among minors. Among adolescents, roughly one in seven high school students have misused opioids at some point in their lifetime, and one in 14 is currently misusing opioids (1). Studies also display that youth ages 18–25 comprised the highest percentage of people (24% or 8.3 million people) who used e-cigarettes or other devices to vape nicotine in 2022 (2). Since the risk of e-cigarette use increases at the age of 18, raising awareness of e-cigarette education before the age of 18 may perhaps be necessary to reduce the risk (3). Additionally, due to the overall lack of education related to opioid and e-cigarette misuse, engaging youth populations in interactive educational interventions was of the utmost importance to bolster their knowledge (4, 5).

The association between substance use among adolescents and high school dropout rates is also concerning. According to Tice (6), 12th-grade dropouts are more likely to engage in illicit drug use than their counterparts enrolled in school. Longitudinally, it has been demonstrated that students who do not use substances such as opioids, nicotine, and cannabis are more likely to have better academic outcomes than peers who are substance users (7). To prevent opioid and e-cigarette use among young individuals, this vulnerable population must first understand how these substances impact their health and why they can lead to addiction, which raises a large discussion regarding health literacy (8). By gaining health literacy, they gain a deeper understanding of evidence-based wellness topics, such as abstaining from or ceasing substance use, which in turn motivates them to make informed decisions (9).

The Substance Use and Prevention (SUP) program, established in 2019 at Michigan State University College of Osteopathic Medicine (MSUCOM), has collaborated with school districts toward health literacy for high and middle school students. SUP program implements comprehensive and dynamic presentations to teenagers from different areas of southeast Michigan (10). In the SUP program, medical students receive high-quality education under the guidance of expert faculty members—including physician internists, pediatricians, and pharmacists/PhD in pharmacology and toxicology—to prepare content and active methods of interactions to present evidence-based knowledge to teens. SUP focuses on providing education for the prevention of opioids, tobacco, and cannabis, including e-cigarettes, while offering opportunities for medical students to interact, enhance their communication skills, and engage in research projects. To foster student leadership in community services and scholarly activity, the program holds a committee led by 2nd, 3rd, and 4th-year medical students engaged in a long-standing approach to substance use prevention, ensuring the program's sustainability and impact over time. SUP student leaders are key players in all process steps, including contacting school administrators, community leaders, and recruiting medical students as volunteers in the interactions. Faculty members play a crucial mentorship role in training these students and ensuring that the program's content adheres to the highest ethical and scientific standards, thus enhancing the overall learning experience for the medical students involved.

These discussions between medical students and adolescents are unique due to their peer-to-peer format (11–13). Our main goal is to provide adolescents with a distinctive skill set for substance use prevention by creating an environment where youth feel safe and utilizing an objective science-based approach through interactive education methods and case-based learning.

Previous literature has shown that school-based awareness is effective when interactive techniques such as break-out groups and Q&A sessions are incorporated to engage students in critical thinking (14–16). Previous interventions have been attempted since the late 1990s and early 2000s, such as the DARE (Drug Abuse Resistance Education) program; however, studies have demonstrated that such programs were ineffective as they failed to be relatable to and interactive with the youth population (11, 12).

SUP serves as a call to action for safety, awareness, and support in addressing pressing public health concerns. The SUP program offers a unique scope for medical students to engage in community activities and extracurricular initiatives, setting it apart from other programs. These opportunities include student-led educational interactions with close contact with high school instructors and principals as a strategy to effectively learn and address the community's needs and tailor substance use prevention messaging to their specific local concerns. Additionally, students are guided to prepare thoroughly for these presentations, applying and reinforcing the knowledge gained in their classroom studies. Since much of the content aligns with their curriculum, including basic sciences and system courses, this process not only supports their academic learning but also deepens their practical understanding and ethical/humanistic development.

We hypothesize that most high school students in our regions of interest were unaware of the causes, health consequences, and financial burden of e-cigarettes and opiates and especially lack knowledge of opioid reversal medications. This study aimed to evaluate the impact of the SUP intervention utilizing evidence-based health literacy of substance use, which may suggest peer-to-peer interactions driven by medical students could serve as a sustainable and effective delivery method.

## 2 Methods

### 2.1 Study design, population, and data collection

This quasi-experimental non-randomized study design followed a pre-intervention (to measure baseline knowledge) and a post-intervention (to measure changes in knowledge). It evaluates the impact of educational interventions on the learning outcomes of high school students about opioids and e-cigarettes' mechanisms of addiction, epidemics, health consequences, and prevention. Students invited to participate in the study were enrolled in health education and social sciences classes, including anatomy, biology, and medical careers discipline. A total of 100 students (ages 14–19) from four high schools attended the educational presentations, completed the pre- and post-intervention questionnaires, and were included in this study. Educational presentations were held from January to March 2023 in four high schools in Southeast Michigan, Wayne and Macomb Counties. School 1 had 20 participants, School 2 had 21 participants, School 3 had 40 participants, and School 4 had 19 participants.

Data were collected and analyzed from subjects with signed informed consent forms. Informed consent was obtained from the subjects and their legal guardians (IRB-approved, STUDY00008018). All subjects in the selected classes were invited to participate in the intervention to promote an inclusive environment. However, subjects with an incomplete consent form and those who did not answer a question on either the pre- or post-intervention questionnaires were excluded from the study.

### 2.2 Intervention procedures

The intervention consisted of presentations comprehensively focused on the toxicology and pharmacology of opioids and e-cigarettes, applying accessible and didactic language. The content of the presentation included the active components of generic and brand products containing opioids and e-cigarettes, as well as epidemiologic data on substance usage among teenagers, the history of substance use, short- and long-term health risks, symptoms of use or overdose, and the financial and societal burden of substance use. The presentation also included prevention strategies such as rehabilitation centers and organizations, resources for quitting, and support networks. The criteria for the relevant educational material were based upon the most current epidemiological data (state and nationwide). We tailored the content to align with the school community's needs to address substance use prevention.

Evidence-based literature was utilized to develop interactive presentation topics to engage high school students and encourage participation. Key resources for content creation included the Substance Abuse and Mental Health Services Administration (SAMHSA), the National Institute on Drug Abuse, and the Resources for Adolescent Health at the Center for Disease Control and Prevention (17–19). The content was delivered through visual charts, radiographic images, case studies, and verified testimonial videos from survivors provided by Families Against Narcotics and SAMHSA, highlighting, for instance, the severe health consequences of e-cigarettes, such as collapsed lungs and respiratory distress.

The content was delivered by three to four medical students supervised by the principal investigator (PI) or faculty member from MSUCOM. Under mentorship of faculty experts in the field, they designed the intervention content using evidence-based literature as mentioned above. Participating medical students completed ethics training through the Human Research Protection Program and passed institutional background checks before engaging with minors. To maintain consistency while delivering interactive content among students from the different high schools, medical students followed a protocol to rehearse and standardize the procedures.

The content was delivered using Google Slides or Microsoft PowerPoint slides on each substance (opioids and e-cigarettes), lasting 45 min and presented in different blocks. We used strategies to engage the high school students during the presentations, such as bingo and other games, to foster dynamic interactions. However, these dynamic actions were not used to collect the data reported in the current manuscript. The only data collection tool to assess our interventions was the pre- and post-intervention questionnaires.

On the day of the interactions, to protect their privacy, the subjects were assigned numbers or letters as codes to pair their questionnaires for data analysis posteriorly. Names only appeared on the consent forms, which were gathered before initiating the interactions and data collection. Before the presentation, subjects used their electronic devices to scan a QR code displayed on a PowerPoint slide to complete an online survey (MSU Qualtrics^®^) about demographics (age, ethnicity, and gender). They answered the multiple-choice pre-questionnaire on the content of opioids and e-cigarettes. Following the intervention, subjects scanned a QR code to fill out the post-intervention questionnaire, which contained the same questions they had responded to in the pre-intervention questionnaire. An example of a question asked: “*What is the long-term effect of vaping? a. Eye pain b. Heart and Lung Diseases c. Nausea d. Multiple Sclerosis*.” No grades, GPAs, credits, disciplinary information, nor other academic information concerning this study were assessed, reviewed, nor shared. The questionnaires or interventions did not alter or influence the subjects' academic standing.

### 2.3 Measures

The completed pre- and post-intervention questionnaires were paired using the identifier codes assigned to each subject. Data from subjects who did not complete either pre- or post-intervention questionnaires or did not answer a question were eliminated from the study. The authors graded the answers based on standardized answer keys. Answers to the pre-intervention questionnaire represent the subjects' baseline knowledge before undergoing the intervention. The percent scores (% correct answers) were calculated by adding the total number of correct answers, divided by the total number of questions, for each subject independently. The change in the knowledge gained was calculated by comparing the correct answers on the post-intervention questionnaire to the pre-intervention questionnaire. The number of subjects who answered a specific question correctly (% individuals who answered correctly) was measured by counting the individuals who answered the specific question correctly, then dividing the number by the total number of respondents. Gender and race of each subject were collected and plotted as the percentage of total participants in this study. Data on the poverty level of each school were based on the school's location and collected using the public database provided by the United States Census Bureau.

### 2.4 Statistical analysis

For statistics analysis, we employed the Chi-square test, paired *t*-test, two-way ANOVA, multiple comparisons, Fisher's LSD test, one-way ANOVA, Kruskal Wallis nonparametric test, and uncorrected Dunn's test with α < 0.05. Statistical analyses (CI95) and graphs were performed with GraphPad Prism 10.2.3 (GraphPad Software, Inc., La Jolla, CA). The statistical difference is indicated when the *p*-value is < 0.05 (*p* < 0.05). The analyzed variables are indicated as percentages on the Y-axis of the graphs, and the absolute numbers of schools (*N*) and subjects (*n*) in each school are described in the legends of the respective graphs and listed here. The total number of schools is designated as *N* = 4. The total number of students from all four schools is *n* = 100, distributed as follows: School 1 (*n* = 20), School 2 (*n* = 21), School 3 (*n* = 40), and School 4 (*n* = 19).

## 3 Results

**Figure 1:**
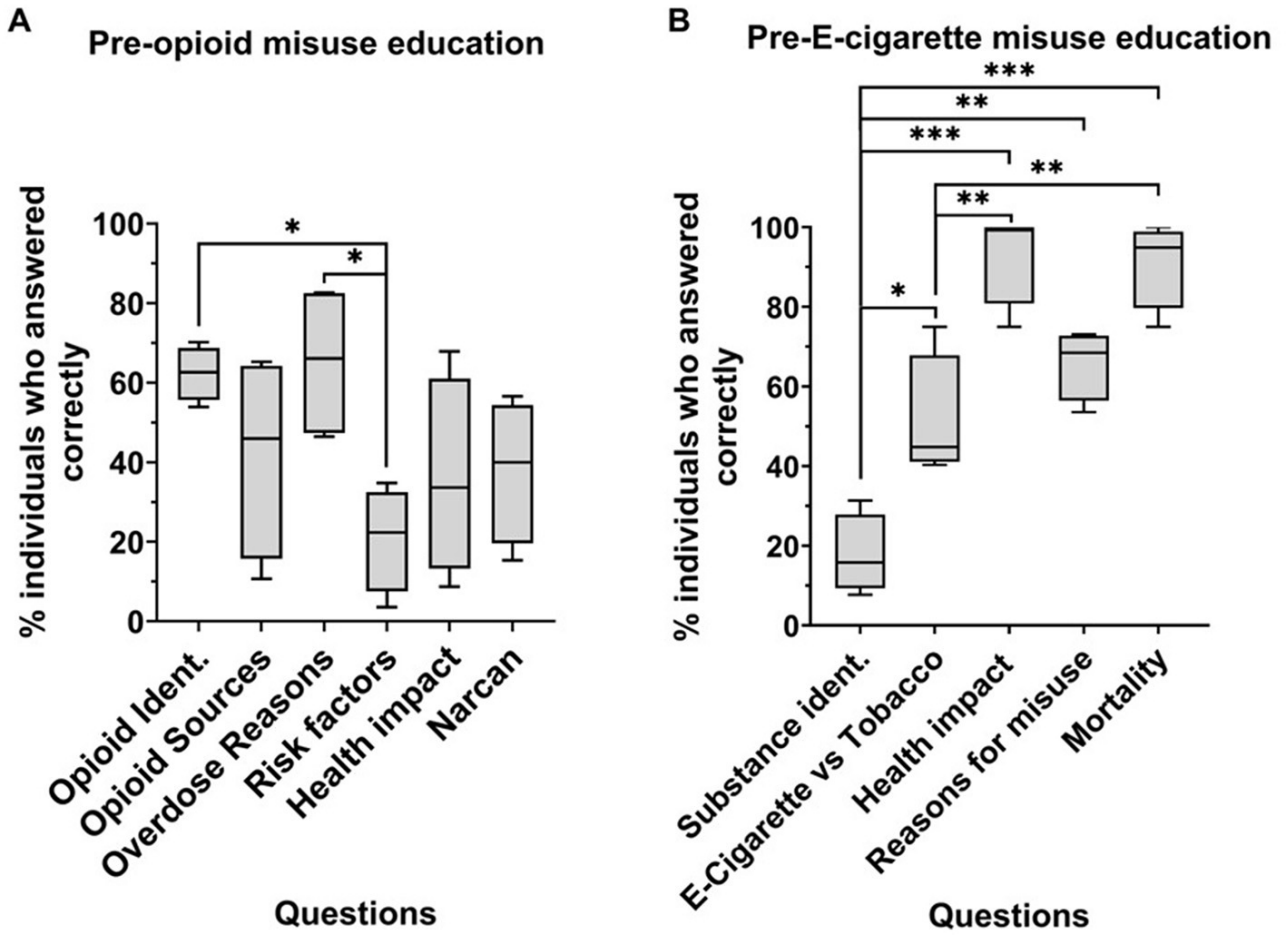
Baseline knowledge on opioid **(A)** and e-cigarette **(B)** misuse across four schools. Percentage of subjects who answered questions related to opioid **(A)** and e-cigarette **(B)** misuse correctly before the intervention. *N* = 4, *n* > 15, school 1 (*n* = 20), school 2 (*n* = 21), school 3 (*n* = 40), school 4 (*n* = 19). Two-way ANOVA, multiple comparisons. *p < 0.05, **p < 0.001, ***p < 0.0001. Opioid ident, opioid identification; Substance ident, e-cigarette identification; e-cigarette vs. tobacco, questions related to comparing the two substances.

One hundred high school students (14–19 years of age) from four schools participated in the study to evaluate the impact of an educational intervention on opioids and e-cigarettes on their learning (Supplementary Figure S1A). The students' baseline knowledge, as represented by the pre-intervention questionnaires, varied among the topics of substance misuse (Figures 1A, B). Sixty-two percent of subjects have higher scores in identifying opioid drug names, and 65% of subjects knew why overdose occurs (“overdose reasons”) more than the risk factors of opioid misuse (21%) (Figure 1A). Eighteen percent of subjects demonstrated difficulty in the identification of e-cigarettes compared to identifying differences between e-cigarettes and tobacco (51%), health impact (93%), reasons for misuse (66%), and mortality (91%), and 51% of subjects demonstrated difficulty in identifying differences between e-cigarettes and tobacco compared to recognizing the health impact (93%) and mortality (91%) (Figure 1B).

**Figure 2:**
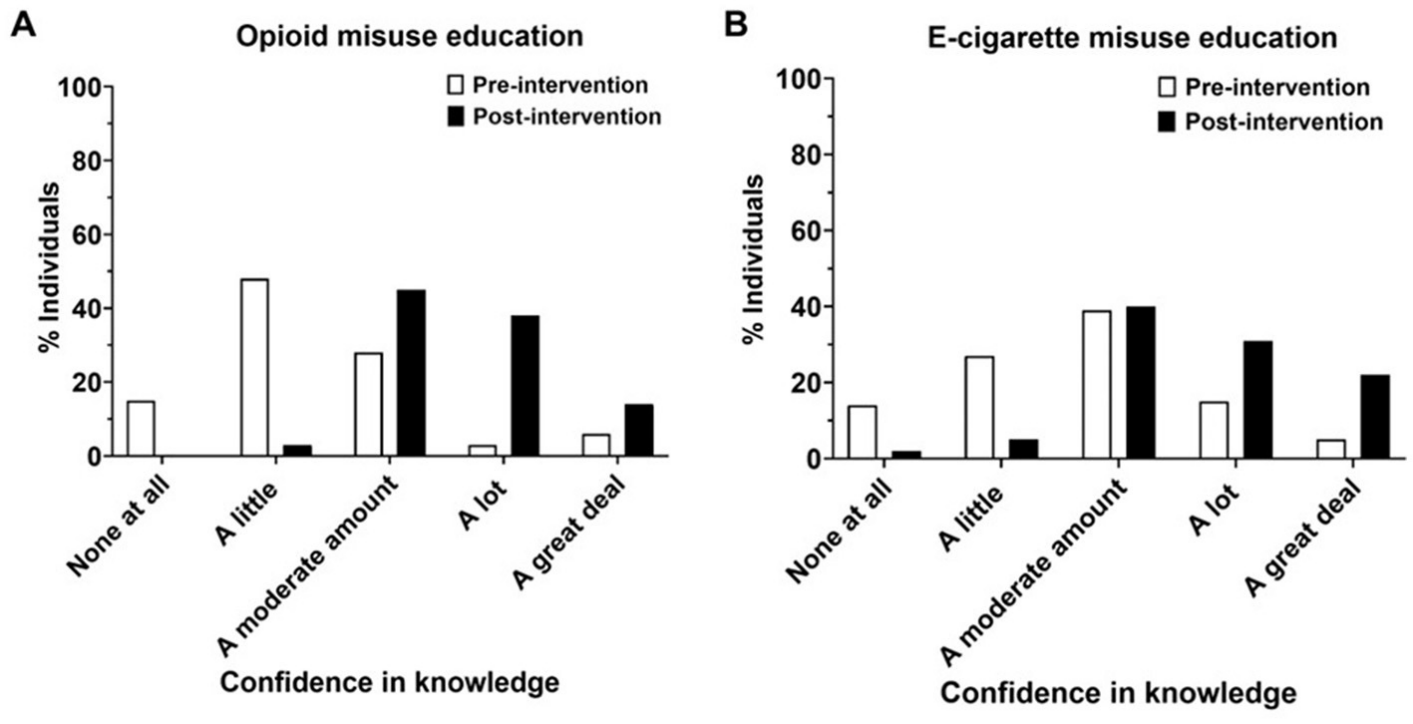
Subjects self-rating their knowledge on opioid **(A)** and e-cigarette **(B)** misuse pre- and post-education in four schools (schools 1–4) *N* = 4, school 1 (*n* = 20), school 2 (*n* = 21), school 3 (*n* = 40), school 4 (*n* = 19). Chi-squared test. *p*-value < 0.0001.

After undergoing the intervention, subjects reported having considerably more confidence in their knowledge of opioids and e-cigarette misuse (Figures 2A, B; *p* < 0.0001). Nine percent of individuals rated their opioid knowledge as “a lot” or “a great deal” pre-intervention, which significantly increased (*p* < 0.0001) to 52% of individuals post-intervention (Figure 2A). For e-cigarettes, 20% of individuals rated their knowledge as “a lot” or “a great deal” pre-intervention, which also significantly increased (*p* < 0.0001) to 53% of individuals post-intervention (Figure 2B).

**Figure 3:**
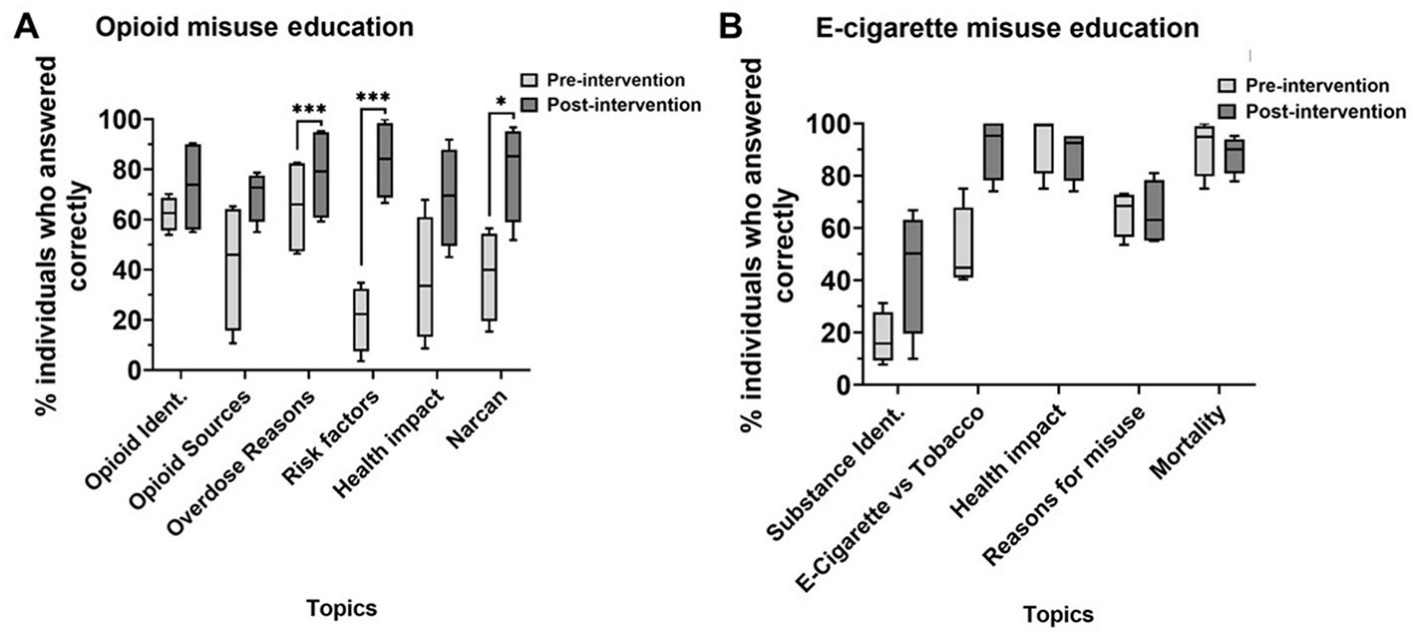
Percentage of subjects who answered questions related to opioid **(A)** and e-cigarette **(B)** misuse correctly pre- and post-education. *N* = 4, school 1 (*n* = 20), school 2 (*n* = 21), school 3 (*n* = 40), school 4 (*n* = 19). *p < 0.05, ***p < 0.0001, paired *t*-tests.

Next, we determined whether the increased reported confidence was consistent with their actual scores on the questionnaire (Figures 3A, B). We showed that subjects significantly improved identification of overdose reasons (65 vs. 78%, *p* < 0.001), risk factors (21–84%, *p* < 0.001), and naloxone as an emergency treatment (38–80%, *p* < 0.05) for opioid misuse education. Regarding e-cigarette education, subjects from all schools combined did not perform significantly better, possibly due to a higher baseline knowledge of topics related to e-cigarette misuse (Figure 3B).

**Figure 4:**
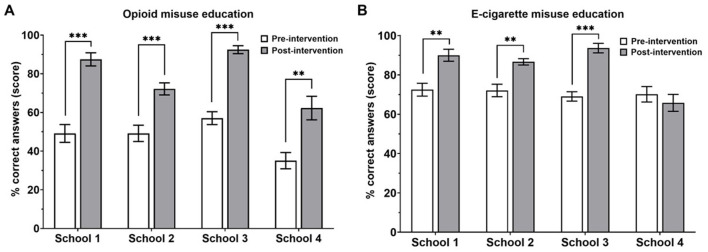
Percent scores on questionnaires related to opioid **(A)** and e-cigarette **(B)** misuse pre- and post-education. School 1, *n* = 20; school 2, *n* = 40; school 3, *n* = 21; school 4, *n* = 19. **p < 0.001, ***p < 0.0001, paired *t*-tests.

To assess the overall education gained following our implemented intervention, we analyzed the difference in subject performance using the correct scores (%) pre- and post-intervention per school. We found a significant improvement (*p* < 0.0001) in overall performance for opioid misuse education in all four schools (Figure 4A), and, except for school 4, our intervention significantly increased (*p* < 0.001) overall performance in e-cigarette education in all the other schools (Figure 4B).

**Figure 5:**
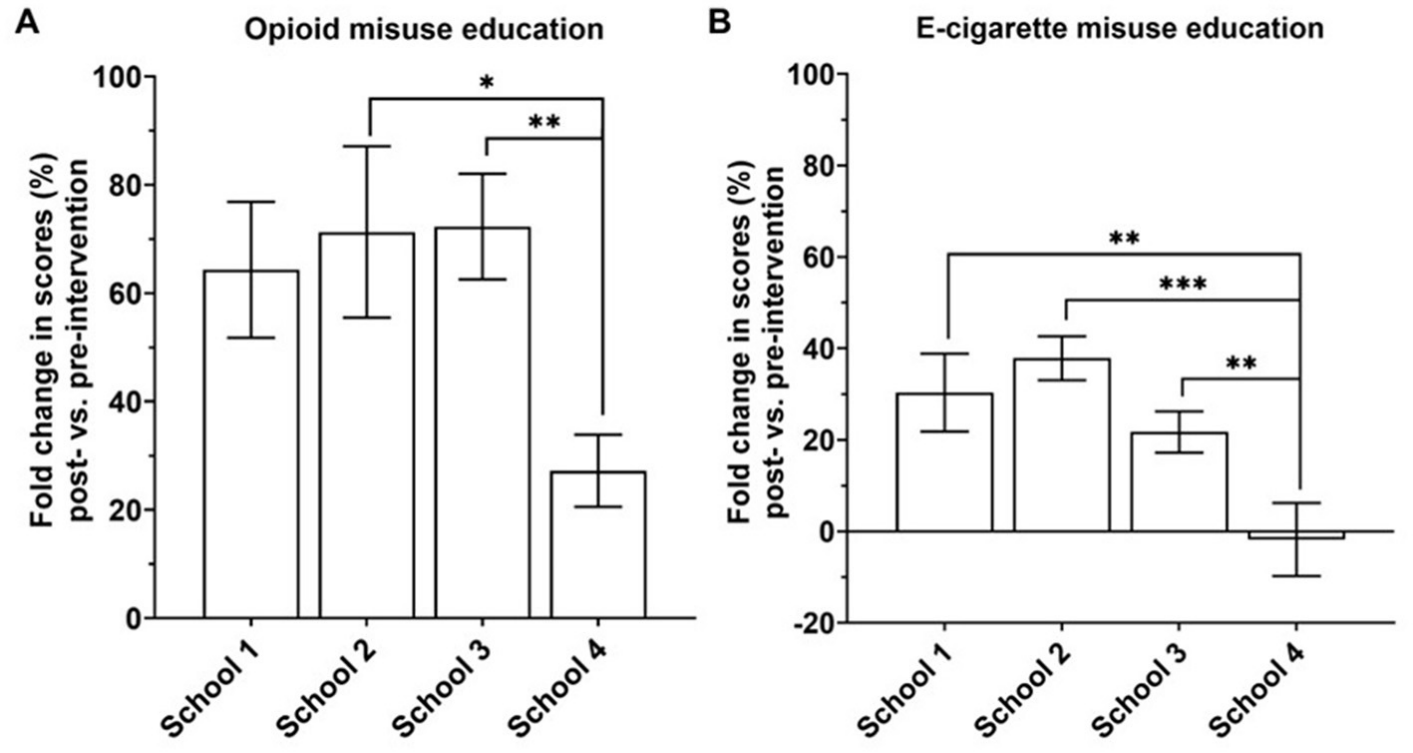
Fold change (%) in scores on a questionnaire related to opioid **(A)** and e-cigarette **(B)** misuse among schools. School 1, *n* = 17; School 2, *n* = 21; School 3, *n* = 38; School 4, *n* = 19. *p < 0.05, **p < 0.001, ***p < 0.0001, one-way ANOVA, Fisher's LSD test.

Next, we aimed to identify factors contributing to the observed variance in school score improvement. Compared to schools 2–3, subjects from school 4 demonstrated the least improvement in opioid education, with only a 28% improvement in scores (Figure 5A, *p* < 0.05). Comparing schools 1–4 resulted in a nearly significant difference (*p* = 0.054). Similar to opioid education, subjects in school 4 performed statistically lower (*p* < 0.001) in e-cigarette learning than those in schools 1–3. Interestingly, subjects from school 4 demonstrated a near 0% change in e-cigarette performance (Figure 5B).

**Figure 6:**
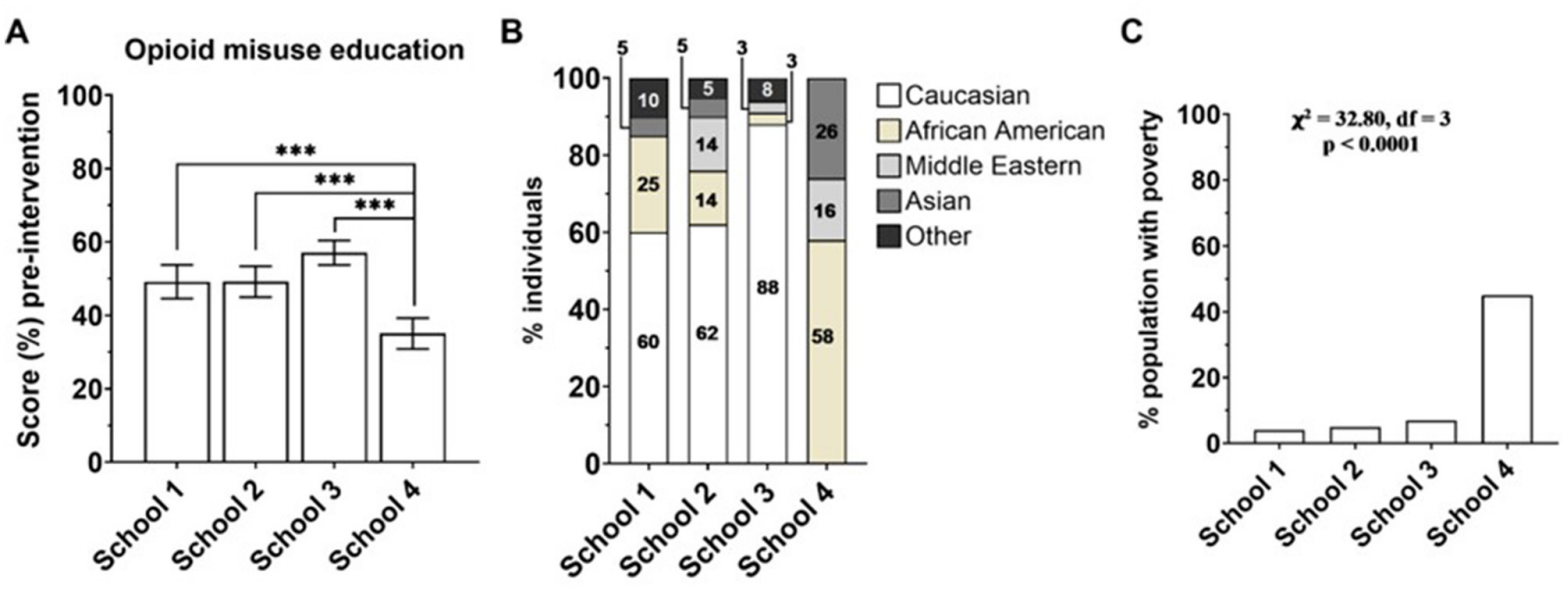
**(A)** Correct answer (%) on a questionnaire related to opioid misuse pre-intervention. School 1, *n* = 20; School 2, *n* = 21; School 3, *n* = 40; School 4, *n* = 19. ***p < 0.0001. One-way ANOVA, Fisher's LSD test. **(B)** Race distribution of subjects at schools 1–4. Chi-squared test of independence, χ^2^ = 58.13, *df* = 12, *p* < 0.0001. **(C)** percentage of the population with poverty (income in the past 12 months below the poverty level) in the zip code location of each school, obtained from the United States Census Bureau (30). Chi-squared test for poverty levels among school zip codes, χ^2^ = 32.80, *df* = 3, *p* < 0.0001.

The differences in scores pre- and post-intervention may be attributed to variations in baseline knowledge of the subjects (pre-intervention). Therefore, we compared baseline knowledge, measured by the percent correct scores on the pre-intervention questionnaire among schools. We found that school 4 had a significantly lower (*p* < 0.0001) baseline in opioid education compared to schools 1, 2, and 3 (Figure 6A). Taken together, the reduction in percent change in scores of school 4, as seen above, is attributed to a significantly low baseline knowledge (Figures 5A, 6A; *p* < 0.05 and *p* < 0.0001, respectively).

Interestingly, despite a baseline education that was not significantly different among schools 1–4 (Supplementary Figure S1B), following the intervention, school 4 significantly declined (*p* < 0.001) in improving in e-cigarette education scores (Figure 5B).

## 4 Discussion

We hypothesized that the prevalence of opioid and e-cigarette crises among young adults might stem from a lack of education on these health topics. To address this, we developed a strategy to assess health literacy followed by an educational intervention conducted by SUP. This intervention aimed to improve adolescents' health literacy and understanding of substance misuse. Our study revealed that participants, before the SUP intervention, lacked an understanding of important information in opioid and e-cigarette misuse (Figure 4). Participants reported significantly higher confidence in their opioid (*n* = 4, *p* < 0.0001) and e-cigarette (*n* = 5, *p* < 0.0001) knowledge after the intervention (Figures 1A, B). They significantly improved their ability to recognize causes of overdose (scoring 65 vs. 78%), risk factors (21–84%), and naloxone as emergency treatment (38–80%) (Figure 3A). However, subjects showed no changes in individual e-cigarette questionnaires, which we attribute to school-specific variances in knowledge and/or high baseline knowledge (Figures 3B, 4B, Supplementary Figure S1B). The difference in knowledge among schools is likely due to racial disparities, and differences in socioeconomic status, as shown by the increased poverty level, further explored later in this section (Figures 6B, C).

Subjects showed a greater baseline knowledge in certain areas of substance misuse than others. Although the students were unfamiliar with the risk factors for opioid use, they were familiar with reasons why overdose could occur, opioid drug names, and an understanding of opioid misuse and its consequences to individuals' overall health. In fact, opioid identification and reasons for overdose may be well advertised on social media better than other sub-topics of the opioid crisis (20). Therefore, advertising may have influenced baseline health literacy and contributed to the observed variability in certain aspects of opioid misuse (Figure 3A).

Interestingly, subjects exhibited a greater baseline knowledge of e-cigarette use compared to opioids. Subjects were knowledgeable about the health consequences, motivations, and factors related to e-cigarette overuse and mortality; nevertheless, they were unaware of the distinctions between e-cigarettes and tobacco.

Similar to opioid knowledge, subjects are more likely to understand the health impact of e-cigarettes, the reasons for misuse, and mortality compared to other topics (21). However, advertising may have been less effective in areas where students demonstrated significantly less knowledge in certain topics, such as understanding the risk factors for opioid use and distinguishing between e-cigarettes and traditional tobacco products. In contrast, students exhibited remarkable understanding of health impacts, reasons for misuse, and mortality, likely due to the prevalence of advertisements addressing these topics.

Public advertisements may lead to intentional bias and gaps in knowledge (21, 22). For example, few advertisements discuss what an e-cigarette is and how it is different from traditional tobacco; in addition, many other studies have shown that these private companies tend to highlight the same information (23). Advertisements also highlight that e-cigarettes are less harmful than their cigarette counterparts, without due explanation (24). A recently published study found increased perceived social benefits, increased friendships, and product-related appeals, such as the innovative design and variety of flavors, were major attractions in advertisements (25). There is a lack of critical discussion of substance misuse regarding e-cigarettes and opioids in their current school curriculums, and social media may not always be promoting accurate materials (22). Future studies are needed to explore the impact of substance misuse (as opposed to promoting usage) advertisements on knowledge and behavior in teens.

In summary, our intervention has significantly contributed to their education, demonstrated by an increased confidence in the acquired knowledge about both opioids and e-cigarettes. Across the other three schools, our intervention significantly impacted opioids and e-cigarettes on the students' education. Interestingly, when comparing score performance among e-cigarette topics in the questionnaire, we did not obtain significant differences before and after the intervention (Figure 3B). However, the intervention had a school-specific impact on their total understanding (Figures 4, 5). Although the educational intervention did not impact students from school 4 in e-cigarette knowledge, it was significantly beneficial for this school to learn about opioids. School 4 may have characteristics that distinguish it from others regarding substance use understanding.

To investigate factors affecting the variance in e-cigarette education of school 4 compared to schools 1–3, we predicted that gender roles amongst the schools may not contribute to the changes seen in performance because, as shown in Supplementary Figure S2, the distribution of genders amongst schools is not significantly different. Importantly, schools 1–4 differed in socioeconomic status and race (Figure 6). We hypothesized that race disparities among schools may have contributed to the differences in performance since our data revealed that race distributions are significantly different—schools 1–3 have a primary Caucasian demographic. In contrast, school 4 has a primarily African American demographic (Figure 6B).

Additional factors, such as parental influence or lack of parental supervision, public health awareness in different regions, and variations in school programs and substance misuse education, may have further contributed to differences in substance misuse knowledge among schools. Future studies could further examine these factors to gain deeper insights into the influences on teens' health literacy. Multiple studies have associated poverty with lower social achievement (26, 27). This is supported by related findings of increased stress in the child's home, food scarcity, and neighborhood violence, all contributing to socioeconomic disparity between students (28). Therefore, we investigated poverty level as another factor that could play a role in e-cigarette education differences among schools and observed a significant increase in the poverty level in school 4 compared to the others. We concluded that the increased poverty level in students from school 4 may play a role in knowledge improvement. Our findings are in line with Harms and Garrett-Ruffin (28) regarding disparity as we detected through data analysis of educational attainment from our presentations comparing schools 1–3 with school 4, which has the highest level of poverty (Figure 6).

This divergence between attainment from those with increased poverty is also paired with adolescents with increased utilization of substance use. Studies show those with an income of < $20,000 are 34% more likely to report substance misuse (29). The data reflects how our presentations, though equal, show a lack of equity in areas with low socioeconomic status. A combination of social factors, such as normalized substance use, high-stress home life, and neighborhood violence, all contribute to perpetuating substance abuse and lead to further denial regarding substance abuse as a problem. The inequity displayed with school 4 displays room for growth in our ability to address an audience that needs our help the most. We have shown that the SUP methodology, which employs active interaction techniques to present evidence-based knowledge, is effective in educating teens about substance misuse and use prevention. Additionally, it provides medical students with valuable opportunities to interact with their community, refine their communication skills, and participate in research projects.

### 4.1 Strengths and limitations

The SUP program's unique strengths include credible medical students who received high-quality training from expert faculty, establishing protocols to ensure implementation standardization and consistency, and using evidence-based literature to create the education intervention. The overall design delivers content through a consistent, evidence-based framework, ensuring reliability and minimizing variability in presentation. This can mitigate biases such as those generated by self-rated knowledge. The study provides opportunities for community engagement and extracurricular activities for medical students. These future physicians are gaining social, communication, and leadership skills founded on humanistic interactions with the community. Self-rated knowledge, a general possible limitation inherent to data collected through surveys, may have a minor or null impact on our findings. This is because we also included objective questions pre- and post-intervention to measure the students' scores according to the material used in the presentation. The questions are clear and strictly aligned with the content, ensuring relevance and focus. While there is a potential for preconceived bias due to students' prior exposure to substance use prevention topics through other avenues, such as social media, this also underscores the widespread importance of the topic and its relevance in diverse contexts.

While the current findings yielded statistically significant results, we encountered limitations. The primary constraint was the restricted sample size to the number of respondents and schools. Our data consists solely of data from a single cohort at a high school in Detroit, with only three additional non-Detroit high schools. Furthermore, a school's self-selection sampling bias may have caused some limiting impact. To ensure more significant equity and representation, the study's scope must be expanded by incorporating a more diverse selection of high schools with varying socioeconomic status and encompassing rural, urban, and suburban settings across Michigan.

## 5 Conclusion and future directions

In summary, subjects showed improvement in the topics presented. The consistency of these trends across schools underscores the effectiveness and reliability of our presentations. It highlights the valuable educational contribution this substance misuse prevention program offers, which might otherwise be overlooked in conventional curricula previously adopted by programs such as DARE (11). Improving subjects' knowledge indicates that our interactive intervention was effective and can be modeled on a larger scale.

In our subsequent research projects, we aim to compare educational attainment and the efficacy of our intervention model among rural vs. urban vs. suburban regions by extending beyond the confines of Macomb and Wayne counties. We aspire to extend our focus to include prospective study designs and encompass other substances, notably alcohol and marijuana, as part of our ongoing efforts to broaden primary prevention initiatives. Additionally, we plan to include middle school students as part of the program to implement an earlier intervention with the possibility of following this cohort over time to address the limitations of a cross-sectional study. This strategic expansion aims to amplify the reach and effectiveness of our program while concurrently fostering heightened awareness. Collaborative partnerships with other medical schools are integral to this endeavor, facilitating the adoption of a similar approach across multiple institutions nationally.

## Data Availability

The raw data supporting the conclusions of this article will be made available by the authors, without undue reservation.

## Supplementary material

Supplementary Figure S1 **(A)** Descriptive statistics of schools 1–4 categorized by race and gender. **(B)** Percent scores on a pre-intervention questionnaire related to E-cigarette misuse education. School 1, *n* = 20; school 2, *n* = 21; school 3, *n* = 40; school 4, *n* = 19. One-way ANOVA, Fisher's LSD test.

Supplementary Figure S2 **(A)** Association of gender identity distribution among the schools where opioid and e-cigarette misuse interventions were conducted. Chi-square test of independence, χ^2^ = 10.73, *df* = 6, *p* = 0.097. **(B)** Gender identity and percent scores on opioid misuse. **(C)** e-cigarette pre-education questionnaires. Female *n* = 70, Male *n* = 24, other gender *n* = 6; One-way ANOVA, Kruskal Wallis nonparametric test, uncorrected Dunn's test.
