# Supplementary figures and images for: A community outreach program enhances education on opioid and e-cigarette misuse among teenagers
Source: Front Public Health. 2025 Apr 8;13:1490166. doi: 10.3389/fpubh.2025.1490166 (PMC12011856; doi:10.3389/fpubh.2025.1490166)

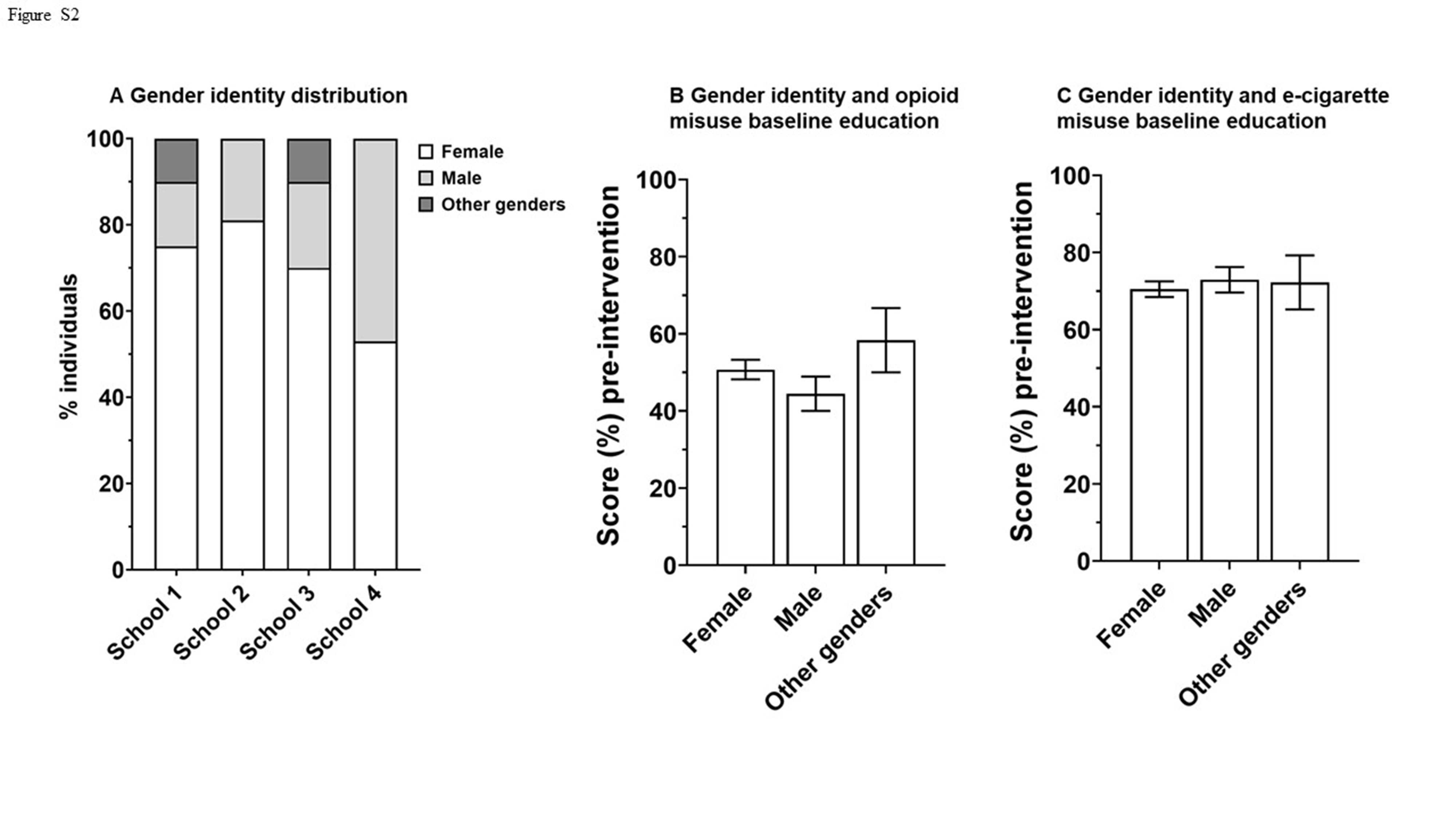

Supplement: Supplementary Figure S1 — (A) Descriptive statistics of schools 1–4 categorized by race and gender. (B) Percent scores on a pre-intervention questionnaire related to E-cigarette misuse education. School 1, n = 20; school 2, n = 21; school 3, n = 40; school 4, n = 19. One-way ANOVA, Fisher's LSD test. [file Image_1.jpeg]

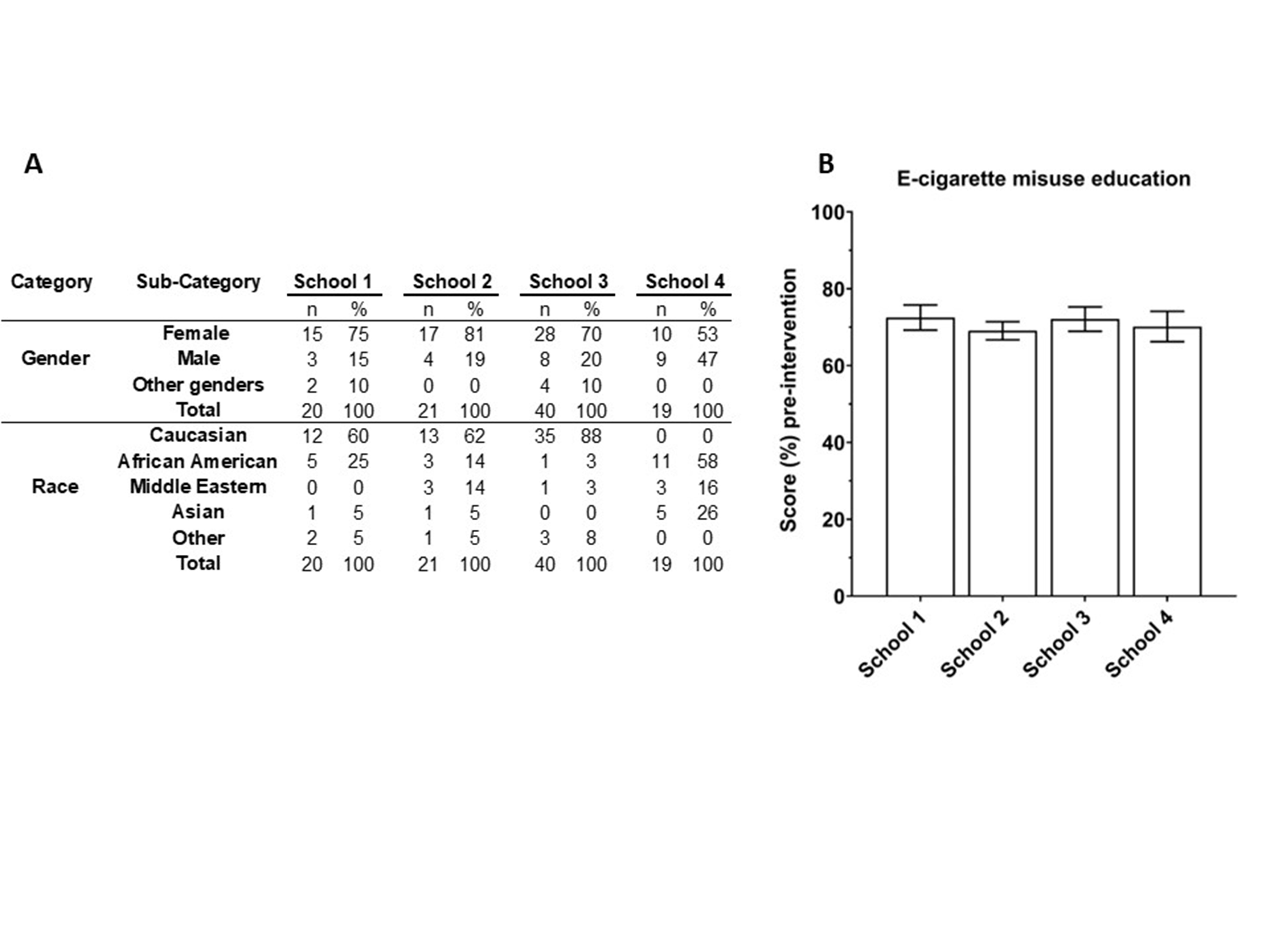

Supplement: Supplementary Figure S2 — (A) Association of gender identity distribution among the schools where opioid and e-cigarette misuse interventions were conducted. Chi-square test of independence, χ2 = 10.73, df = 6, p = 0.097. (B) Gender identity and percent scores on opioid misuse. (C) e-cigarette pre-education questionnaires. Female n = 70, Male n = 24, other gender n = 6; One-way ANOVA, Kruskal Wallis nonparametric test, uncorrected Dunn's test. [file Image_2.jpeg]
